# Acupuncture for perimenopausal insomnia: A systematic review and meta-analysis protocol

**DOI:** 10.1097/MD.0000000000011083

**Published:** 2018-06-15

**Authors:** Taipin Guo, Man Jia, Yuhao Jin, Na Xu, Tianzhong Peng

**Affiliations:** a Yunnan University of Traditional Chinese Medicine, Kunming; b The People's Hospital of Wenshan Prefecture, Wenshan, Yunnan Province; c Nanchang Hongdu Hospital of Traditional Chinese Medicine, Nanchang, Jiangxi Province, China.

**Keywords:** acupuncture, perimenopausal insomnia, protocol, systematic review

## Abstract

**Background:**

Perimenopausal insomnia (PI) is one of the most common complaints in women. Acupuncture is used to treat PI increasingly considering its less side effect. The subject of this study is to explore the effectiveness and safety of acupuncture for PI.

**Methods:**

All the randomized controlled trials(RCT) literatures of acupuncture for PI will be searched in the databases of MEDLINE, Cochrane Library, Web of Science, EMBASE, Springer, WHO International Clinical Trials Registry Platform (ICTRP), China National Knowledge Infrastructure (CNKI), Wan fang, Chinese Biomedical Literature Database (CBM), Chinese Scientific Journal Database (VIP), and other available resources using the subject terms of "acupuncture” and “perimenopausal insomnia” and their synonyms. The languages are limited as English and Chinese. Non-RCT literatures will be screened and relative information will be extracted. Sleep quality values is considered as the primary outcome. Secondary outcomes include biochemical indicators, such as hormone levels, side effects caused by acupuncture, total scores on the insomnia severity index and traditional Chinese medicine symptom changes.

**Results:**

This systematic review study will provide an evidence of acupuncture for PI.

**Conclusion:**

The study will give an explicit evidence to evaluate the effectiveness and side effects of acupuncture for PI.

**PROSPERO registration number:**

CRD42018092917.

## 1 Introduction

### 1.1 Description of the condition

Perimenopause is deemed to a specific surrounding period of the final years of reproductive life, and it starts from the first menstrual irregularity and concludes after 1 year of amenorrhea.^[1]^ Insomnia is a common syndrome in perimenopausal women. According to reports, the prevalence of sleep disorders in perimenopausal women is in connection with culture and ethnicity.^[2]^ The prevalence is high in China, with 51% to 55%.^[3,4]^ It is 40% in Caucasian,^[5]^ 31% to 42% in America,^[6]^ while 28% in Japanese and 15.9% in Korean.^[7]^ Insomnia dose not only have an impact on mental health but also the morbidity and mortality of cardiometabolic and neurocognitive, and it increases health care expense.^[6,8,9]^ Meanwhile, perimenopausal insomnia (PI) is often in a tangle with other coexistent medical conditions such as depression, hot flashes, fatigue, decreased/increased appetite or weight loss/gain, nocturia,^[1,10,11]^ which may amplify harm in relation to common insomnia. The pathological mechanism is not explicit, and study shows lower estradiol and higher luteinizing hormone levels are significantly correlated with PI.^[12,13]^ Also menopausal hormone replacement therapy is widespread used for PI.

In order to obtain high quality of life of menopausal women, adding to menopausal care programmes is necessary during reducing sleep condition.^[14]^ Women suffering from PI also would like to remit their sleep difficulties through comprehensive counseling under the assessment of their body constitution rather than simply prescribing drugs for sleep difficulties.^[15]^ So, it is better that interventions could relieve more symptoms not only insomnia.

### 1.2 Description of the intervention

Various interventions are shown positive effect to improve sleep disorder in menopause. Basing on a literature evaluation of 76 articles,^[16]^ it suggested hormone therapy, isoflavones, escitalopram, gabapentin, eszopiclone, valerian, exercise, and hypnosis to treated insomnia in menopause, and zolpidem, citalopram, quiteiapine XL, mirtazapine followed by long-acting melatonin, ramelteon, Phyto-Female Complex, Pycnogenol, yoga, and massage also could be considered. Eszopiclone could improve insomnia and other symptoms like depressive and anxious, hot flashes in perimenopausal and postmenopausal women.^[17]^ Escitalopram could reduce insomnia symptoms and improved subjective sleep quality in menopausal women with hot flashes.^[18]^

Besides, more and more patients are employing the nonpharmacological alternative therapies and traditional Chinese medicine (TCM), such as acupuncture,^[19]^ Qigong,^[20]^ and Guasha,^[21]^ which have been used nearly 3000 years in China. Research shows acupuncture could decrease the Pittsburgh Sleep Quality Index (PSQI) and change the insomnia severity index (ISI), and polysomnography (PSG) exam show the sleep efficiency and total sleep time are improved significantly after acupuncture treatment.^[19]^

Acupuncture is a therapy that uses a sterile needle to penetrate a specific acupoint in body to treat specifc disease. There are 361 acupoints belonged to 14 main meridians. The acupuncture prescription is consisted of more than one acupoint that is selected based on the complicated TCM theory. The significant advantages of acupuncture are less side effect and significant effect.

### 1.3 How the intervention might work?

According to TCM cognition, PI is caused by the deficiency of liver and kidney, and the mind cannot be nourished adequately coupling with the attenuation of reproductive function. To treat PI, the acupoints that have the function of nourishing liver and kidney and calming the heart and tranquilizing the mind are always been selected. The mechanism of acupuncture for PI is not entirely clear. Research shows that acupuncture could regulate neurotransmitters in the brain like immune cytokines, antioxidant defense systems, hormones, and neuroelectrophysiology,^[22]^ and which may be the reason of the improvement of sleep by acupuncture. Besides, acupuncture also could affect the estrogen receptor expression and then to adjust the estrogen level.^[23]^

### 1.4 Why it is important to conduct this review?

Although acupuncture is widely used to treat PI, and RCT has proved its effect,^[19]^ it still lack of high quality evidence to convince more physician to adopted this treatment.^[16,24]^ The evidence of system review is the highest level, and could give the effect and effectiveness and safety of one therapy for a disease. So, it is important to conduct this study.

### 1.5 Objectives

The aim of this systematic study is to evaluate the effectivity and safety of acupuncture on PI, which may provide evidence to clinician and researcher.

## 2 Methods

### 2.1 Study registration

PROSPERO systematical review protocol registration number is CRD42018092917. This protocol should be reported basing on the Preferred Reporting Items for Systematic Reviews and Meta-Analyses Protocols (PRISMA-P) statement guidelines.^[25]^

### 2.2 Inclusion criteria for study selection

#### 2.2.1 Types of study

In order to estimate the effectiveness and safety of acupuncture on PI, all relevant RCTs will be retrieved and information will be evaluated. This review will include only RCTs about acupuncture on PI. Chinese and English are defined as language restrictions. Others like case reports, animal mechanism studies, non-RCTs, or RCT protocol will be excluded.

#### 2.2.2 Types of participants

There is a restriction on perimenopausal woman. The insomnia may be related to menstrual disorders or unknown causes. The definitions of diagnostic criteria of PI with or without other menopause symptoms will be included.

#### 2.2.3 Types of Intervention

The study focuses on clinical trials of PI with the therapy of acupuncture, and the results will give recommendations to physician. So, different types of acupuncture interventions including manual acupuncture, ear acupuncture, floating needle, electro-acupuncture, etc. will be covered. Both the comparision of acpuncture with other treatment methods and the comparision among different acupuncture methods will be included. Combining therapy which cannot judge the effect of acupuncture will be excluded.

#### 2.2.4 Types of outcome measures

The primary outcome is sleep quality values. Secondary outcomes include biochemical indicators, such as hormone levels, side effects caused by acupuncture, total scores on the insomnia severity index, and traditional Chinese medicine symptom changes.

### 2.3 Data sources

The English databases incorporating MEDLINE, EBASE, Cochrane Library, Springer, WHO International Clinical Trials Registry Platform (ICTRP), as well as the Chinese databases like CNKI, Wanfang, CBM, and VIP will be searched normatively according to the rule of each database.

### 2.4 Search strategy

The following intervention subject term or combination of keyword as acupuncture (e.g., “acupuncture” or “TCM acupuncture” or “ “electroacupuncture” or “fire needling”) will be combined the disease subject term or combination of keyword as PI (e.g., “Climacteric insomnia” or “perimenopausal insomnia” or “Climacteric sleep disorder,” or “perimenopausal sleep disorder” The search strategies for Medline are listed in Table 1.

**Table 1:**
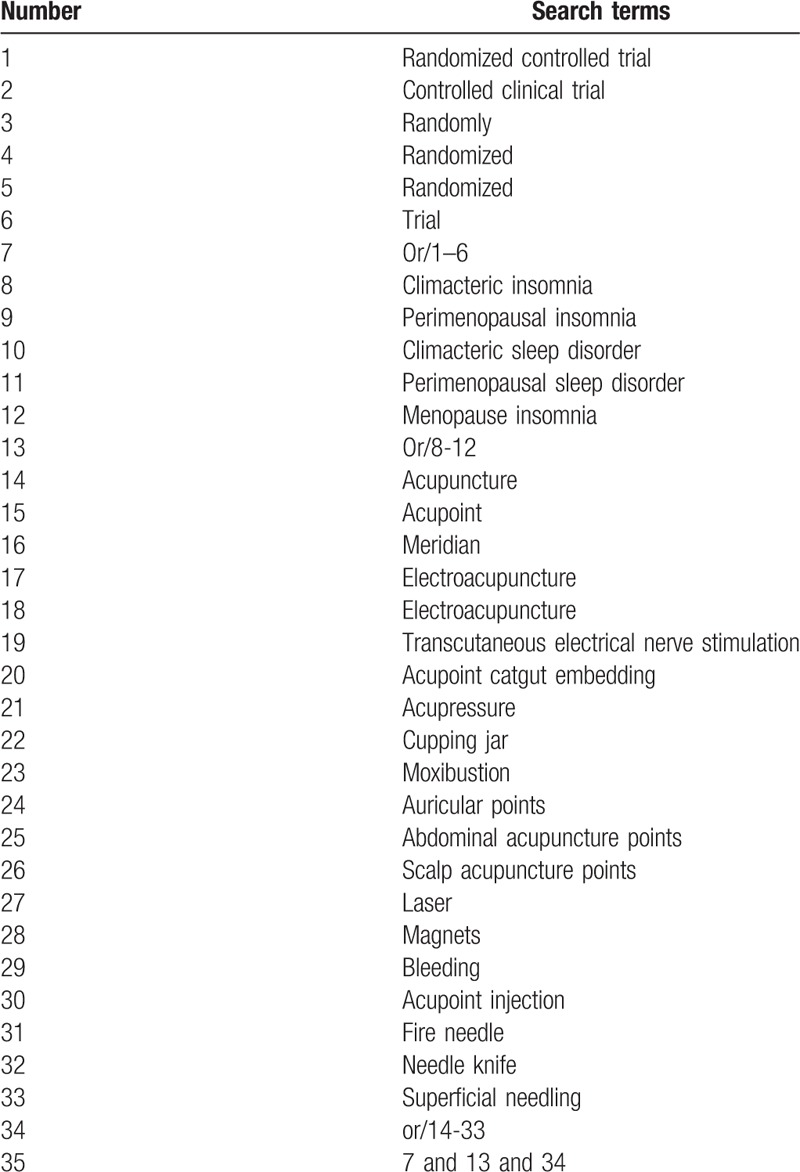
Medline search strategy.

### 2.5 Data collection and analysis

#### 2.5.1 Selection of studies

**Figure 1:**
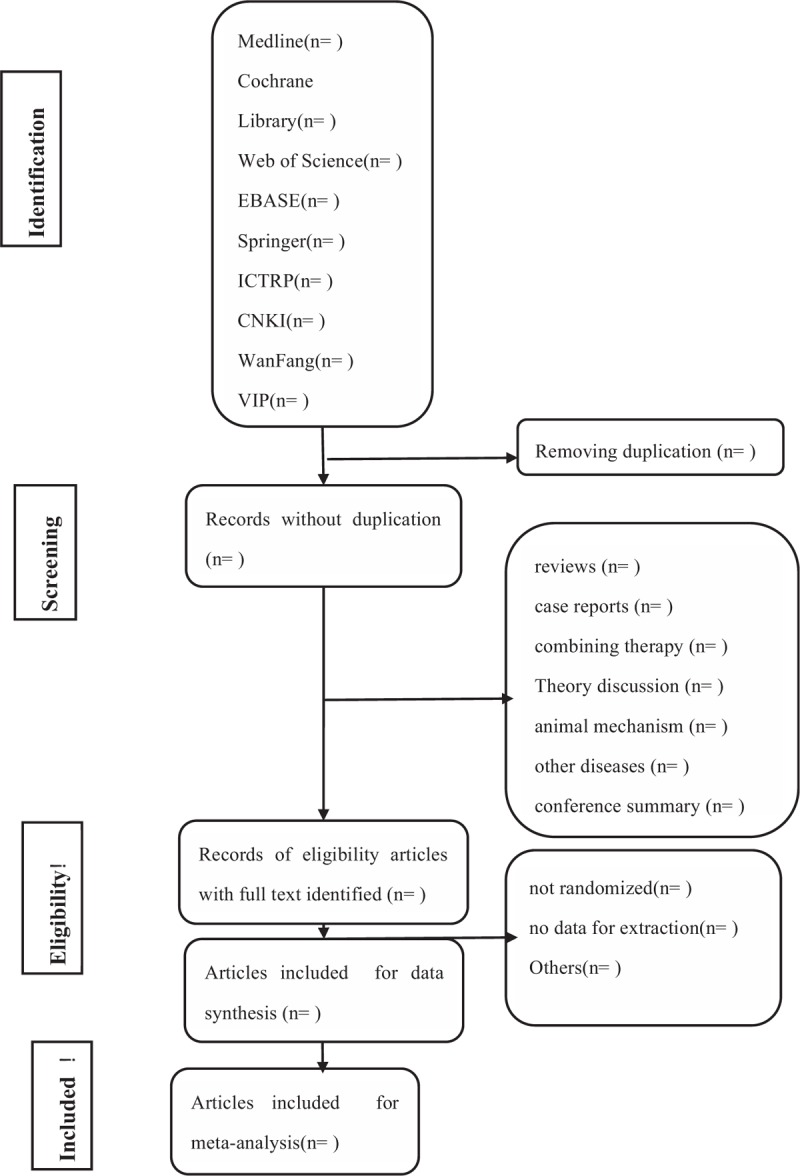
Flow diagram of studies identified.

Two researchers (MJ and YHJ) will independently perform as selection, data extraction, and quality assessment. All relevant articles of full text will be filtrated. When different opinions generate between the 2 reviewers and cannot agree on through consultations, the third reviewer (TPG) will make the final decision. The flow process of filtration is shown in a PRISMA flow chart (Fig. 1).

#### 2.5.2 Data extraction and management

The data such as study design, participant characteristics, interventions both acupuncture, and the control intervention, results will be extracted and recorded in an electronic text. The extraction will be completed independently by 2 reviewers (YHJ and NX) and the information will be recheck crossly. Divergence will be made by the third author (TPG) though discussion.

#### 2.5.3 Assessment of risk of bias and reporting of study quality

To assess the risk of bias, we will adopt the Cochrane risk of bias tool and complete the STRICTA checklist. Meanwhile, the Jadad scale will be employed to estimate the methodological quality.

#### 2.5.4 Measures of treatment effect

Mean differences (MDs) with 95% confidence intervals (95% CIs) will present as the continuous data. Also risk ratio (RR) will be the expression of dichotomous data.

#### 2.5.5 Unit of analysis issues

According to the outcomes, sleep quality values will be pooled to together, and the secondary outcomes including biochemical indicators, total scores on the insomnia severity index, and traditional Chinese medicine symptom changes also be analysed, respectively.

#### 2.5.6 Management of missing data

For missing or incomplete data, we will attempt to contact the original author. Deformity data will be gotten rid of if cannot be supplemented.

#### 2.5.7 Assessment of heterogeneity

χ^2^ test will be applied to calculate the heterogeneity, and the presentation of heterogeneity degree is depended on the *I*^2^ value. According to the results, unimportance of heterogeneity may be explained when the value of *I*^2^ is 0% to 40%, and it exists moderate heterogeneity with the *I*^2^ is 30% and 60%. Meanwhile, it is presented as substantial heterogeneity if *I*^2^ is 0% to 0% and *I*^2^ is 75% to 100% means considerable heterogeneity. The fixed-effect model will be used if *I*^2^≤50% and *I*^2^>50% the random effects model will be chosen.

#### 2.5.8 Assessment of reporting biases

Funnel plots will be used to evaluate the reporting biases when more than 10 trials are included. Its symmetry will account for the biases. There are no reporting biases if the funnel plots are symmetrical, and dissymmetry means it exist biases.

#### 2.5.9 Data synthesis

Quantitative analysis will be implemented using RevMan version 5.3 with 95% CI. The mean change in each of the primary and secondary outcomes will be merged. Besides, if the data does not suit to quantitative analysis, the qualitative description will be employed.

#### 2.5.10 Subgroup analysis

Subgroup analysis will be conducted according to the difference of acupuncture forms, participant conditions and controls.

#### 2.5.11 Sensitivity analysis

We will perform a sensitivity analysis according to the heterogeneity and predefined criteria.

## 3 Discussion

Insomnia is gravely tormenting perimenopausal women and reducing the quality of life. Acupuncture is a nonpharmaceutical therapy that appeals to more and more patient although the action mechanism is not absolutely known, and it almost become a routine treatment replacing the supplement of estrogen in China.^[23,26]^ Insufficient evidence is the restriction of worldwide application. Although the potential low quality of original RCT may influence the reliability of this systematic review, it is still meaningful to carry out this study. This systematic study will merge all the RCT about different kinds of acupuncture stimulation for insomnia in premenopausal women written in Chinese and English, which could provide the efficacy and safety.

## Author contributions

**Data curation:** Tainpin Guo, Yuhao Jin, Na Xu.

**Formal analysis:** Man Jia.

**Investigation:** Na Xu.

**Methodology:** Tainpin Guo, Na Xu.

**Resources:** Yuhao Jin.

**Software:** Tainpin Guo, Man Jia, Na Xu.

**Supervision:** Tainpin Guo, Tianzhong Peng.

**Validation:** Tianzhong Peng.

**Writing – original draft:** Tainpin Guo.

**Writing – review & editing:** Tainpin Guo.
